# Contribution and legacy: a qualitative study of older people’s attitudes about sharing their routinely collected health data for research purposes in New Zealand

**DOI:** 10.1186/s12910-025-01212-6

**Published:** 2025-05-30

**Authors:** Engelina Groenewald, Jasmine Appleton, Brendan Hallam, Cristian Gonzalez-Prieto, Susan Yates, Daniel Wilson, Gillian Dobbie, Rosie Dobson, Sarah Cullum

**Affiliations:** 1 Department of Psychological Medicine, University of Auckland, Auckland, New Zealand; 2 Otago Medical School, University of Otago, Dunedin, New Zealand; 3 Dementia Auckland, Auckland, New Zealand; 4 School of Computer Science, University of Auckland, Auckland, New Zealand; 5 School of Population Health, University of Auckland, Auckland, New Zealand

**Keywords:** Ethics, Routinely collected health data, Health information, Data sharing, Consumer perspectives

## Abstract

**Background:**

Older adults, especially those with dementia, are often excluded from health research due to physical and medical comorbidities, and the assumption that those with cognitive impairment won’t be able to consent. Using routinely collected data for research purposes is a way to include older people in research, and therefore the benefits of research. However, very little research has been done to examine the attitudes of older people towards sharing their routinely collected health data for research purposes.

**Method:**

Twenty-eight semi-structured interviews were conducted with older health service users in the Counties Manukau health district of Auckland, New Zealand. The interviews explored participants’ views around the use of de-identified health data for health service improvement and health services research. Data were analysed using thematic analysis.

**Results:**

Themes identified were: 1) Benefits: participants believed that there were benefits to sharing their health data such as helping others, improving health services, advancing scientific knowledge, and giving back to the health system; sharing health data was also seen as a reflection of good character, and people felt that their pre-existing views about whether they wished to share health data should be respected even if they were no longer able to consent. 2) Concerns: participants had concerns about sharing data with private companies, the use of inaccurate data, and the potential personal and societal consequences of sharing health data. 3) Expectations: participants encouraged collaboration between institutions in New Zealand, but expected data privacy to be maintained, processes to be transparent and cultural values around data to be respected; there was an expectation those sharing health data (patients or institutions) should benefit from any private sector gains.

**Conclusion:**

Older people in our study were supportive of sharing their deidentified health data for research purposes provided that the research would benefit others, now and in the future. This provides more confidence in the use routinely collected health data of older people for research, provided that researchers handle data in a respectful way and use it to benefit communities while avoiding potential harms.

**Trial Registration:**

NA.

**Supplementary Information:**

The online version contains supplementary material available at 10.1186/s12910-025-01212-6.

## Background

Older adults, especially those with dementia, are often excluded from health research. There are multiple reasons for this, including arbitrary age cut-offs, medical comorbidities as exclusion criteria, and the perception that older people may not have capacity to consent or that they will not wish to participate in research [1].

An alternative to actively recruiting participants for research is using routinely collected health data for health research. This helps to reduce selection bias by including older people, thus making the findings more generalizable to this group and allowing them to benefit from the generated research evidence. Some argue that not using routinely collected health data potentially causes harm due to the consequent lack of robust evidence for benefit or harm in people who are unable to participate in traditional research. This further perpetuates the impact of ageism, stigma and discrimination [2, 3]. Using routinely collected health data may also be more inclusive for those with cognitive impairment who are often excluded from traditional research projects due to researchers’ assumptions about capacity to consent [1].

Using routinely collected health data for research in older adults has many potential applications such as falls prevention [1], and the prevention and management of chronic diseases [4]. In the area of dementia, routinely collected health data could be used to help improve early diagnosis, identify biomarkers, prevent decline in brain health, and monitor progression of dementia [5–7].

The utilisation of administrative data is increasing rapidly due to the explosion of artificial intelligence (AI) and is likely to be a cost-effective approach research tool for epidemiology and dementia prevention studies. The development of AI in health care [8] and the future applications of this technology are anticipated to particularly benefit older people as they are the highest users of healthcare services [9].

Despite the apparent advantages of using deidentified routine data, there is a prevailing perception that data belong to individuals, and we therefore need to understand how people feel about the use of their health data for research purposes. Surveys exploring people’s attitudes and perceptions regarding this issue [9, 10] have shown that people are generally positive about the use of their data for research, but oppose the idea of their data being shared with private companies [11–14]. Qualitative research studies have used one-to-one interviews and focus groups to examine these issues in greater depth, exploring patients’ attitudes towards the sharing of routinely collected health data. Several studies [2, 15, 16] reported that participants were generally positive towards data sharing due to its potential benefits for patients, the health community, and health services research. However, patients were concerned about confidentiality and potential misuse of their health data, for example, to discriminate or restrict their access to specific health services, and also potential discrimination by insurance companies.

Very few studies have explored older people’s attitudes and preferences around sharing their routinely collected health data for research purposes, especially in the context of brain health. One survey conducted in the United Kingdom reported that, of the whole population surveyed, people in the age group aged 75 + were the most willing to share their health data [13]. Another survey of older adults attending senior centres in South Korea, showed that older adults were willing to share health data with families and hospitals but less willing to share with researchers and device developers [4]. An online survey of older outpatients in New Zealand specifically addressed the issues of cognitive decline and dementia and found that patients supported the use of their data provided that the population would benefit from its use [17].

Whilst these survey results are important, the findings need to be investigated in more depth to understand the nuances, especially for the population at higher risk of dementia due to their age. To our knowledge there have been no qualitative research studies using one-to-one interviews or focus groups that specifically recruited older people to explore their attitudes about sharing their health data. The aim of this study was therefore to explore older people’s attitudes and preferences about sharing their routinely collected health data for research purposes, especially in the context of brain health.

## Methods

### Population and setting

The study took place in Te Whatu Ora Counties Manukau, a health district in Auckland, New Zealand, with a highly diverse population including Māori, Pacific Island, Asian and European peoples. The sample was ascertained from a subsample of service users who responded to a prior online survey which sought to gain an understanding of the attitudes of older people about the management of their health information [17]. The survey was distributed by email to people who met the following inclusion criteria: [1] currently resident in New Zealand [2] aged 55 years or older, and [3] currently using health services in Te Whatu Ora Counties Manukau. All 326 service users who participated in the online survey were asked if they would be willing to be contacted for a further face-to-face interview to obtain more in-depth knowledge about the attitudes and preferences around the use of their health information, and 84 people agreed to be contacted.

### Procedures

We contacted the survey participants with correct contact details and asked them whether they were still interested in participating in an online interview to be conducted in English, and 28 participants agreed. Semi-structured in-depth interviews were conducted by a trained female research assistant (JA) using the Zoom video conferencing platform. We had originally planned to conduct face-to-face meetings but were unable to do this due to policy around COVID-19 restrictions at the time. Interviews were audio-recorded, and transcribed using a package for automatic speech recognition called Whisper, developed in Python [18]. Whisper was chosen for confidentiality reasons, as it does not connect to other servers online.

### Interview guide

The research assistant (JA) used a case scenario to guide the interview. The scenario was based on a previous more generic case scenario developed by one of the authors (RD) for a similar study in younger adults [2]. Our case scenario explored topics around the use of de-identified health data for health service improvement and health services research, but with a specific focus on brain health and dementia. The topics included sharing data with academic partners local and overseas, linkage with other health data (e.g., utilisation of healthcare resources, mortality data); allowing private companies to use the data in order to develop new medical equipment (possibly for profit); and using data from people who are unable to consent (such as those who have developed severe dementia or have died). Participants were encouraged to ask questions and discuss each step of the scenario so that they fully understood the issues. The topic guide can be found in Additional File 1.

### Analysis

The qualitative study used an inductive approach. Transcripts were analysed by two of the authors (BH and LG) using thematic analysis. The phases outlined by Braun and Clarke [19] were used for analysis. Thematic analysis was chosen as a method to analyse the data, as it enabled the investigators to understand the motivation for participants’ attitudes towards sharing health data, and the meaning those attitudes had for them. The two team members familiarized themselves with the data, documented initial ideas and themes, and then started data coding across the whole dataset. The case scenario questions helped shape how data was collected, but the researchers didn’t use those questions as a predetermined coding framework for their analysis. The initial codes were collated into potential themes and were cross-checked with coded extracts and the data set. The two researchers (BH and LG) had regular meetings where themes were reviewed, defined and named to produce the report. After the themes were identified, the researchers went back to the transcripts to find exemplars.

### Ethics

Ethical approval for this study was obtained from the Auckland Health Research Ethics Committee, reference number AH22266 on 18 October 2021.

## Results

### Sample characteristics

Online interviews were conducted with the 28 participants who agreed to take part in the study. Most (26/28) of the participants were aged 65+, the remaining two were 57 and 60 years old. Ages ranged between 57 and 90, with an average age of 74 years. Of the 28 participants, 25 were of European ethnicity, one participant was Māori, one was Fijian Indian, and one did not disclose ethnicity. Nineteen participants were female and seven were male. Six participants stated that they had personal experience of living with or caring for a person who had been diagnosed with dementia.

### Thematic analysis

Participants were generally positive about their routinely collected deidentified health data being used for research purposes. They perceived many benefits to sharing their health data such as helping others, improving health services, advancing scientific knowledge, and giving back to the health system. They felt that sharing their data was a reflection of good character, and that people’s pre-existing views about whether they wished to share health data should be heard and respected, including if they were no longer able to consent.

They also voiced the concerns they had, such as sharing data with private companies, sharing inaccurate data, and the potential personal and societal consequences of sharing health data.

Participants had strong opinions on how research should be conducted, including an expectation that institutions in New Zealand should collaborate to get better results. They expected data privacy to be maintained and processes to be transparent. Some felt that patients and/or institutions should benefit from any private sector gains, and that cultural beliefs should be respected.

**Fig. 1:**
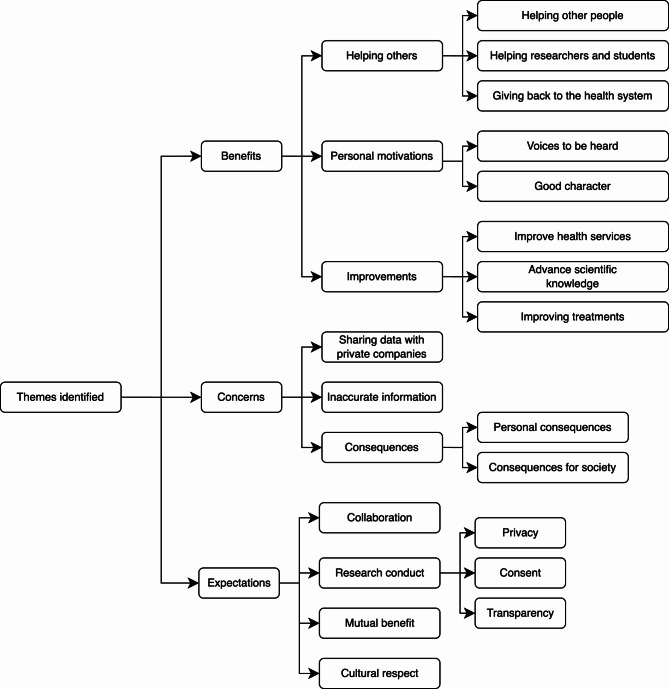
Key themes identified by older people around the sharing of deidentified health data

The main themes and subthemes are illustrated in Fig. 1.

### Benefits to using routinely collected health data for research

Participants were willing to share their health data for research purposes if there was a perceived benefit to doing this in order to help others, for themselves, and to improve health services. It was also seen as a way to give back to health services that had served them over their lifetime.

Participants wanted to aid, assist and support others by sharing their data. “Others” refer to people in general, but also specific groups such as students or researchers, and even institutions like the hospital. The idea of helping the health system by “giving back” was included in this subtheme, as participants were the recipients of help and support from the hospital, and therefore wanted to provide aid to the health system in return.

### Helping other people

The foremost reason for being willing to share data was the potential to offer help to others, in particular people living in the local community.

*I don’t have any difficulties with that (sharing health data) because if I can help other people*,* I love to do that [Participant 21]*.

*I’d be very happy (to share health data). I think anything that can help is wonderful. [Participant 13]*

*I’m okay with it being used for research because it’s only going to help people…possibly not myself*,* but certainly other people. So why not? [Participant 26]*

In addition to helping other people now, participants also saw sharing of their health data as a way to help other people in the future. One participant used migraine as an example and said that he would be more than willing to share his health data if it could help one of his children in the future. Another pointed out that increasing prevalence of dementia was likely to have a major impact on the health system, so obtaining knowledge now would benefit those in the future.

*My understanding of what I have read: in the next*,* I forgot how many years but it’s not that long*,* one in three people are going to end up with dementia. If you know those statistics are correct*,* well*,* that’s a major. It’s a major on the health system. It’s a major on families. It’s a major on doctors*,* everything. So there’s got to be a reason why more people are getting dementia. It’s going to be one in three. You know*,* that’s really high. So what is the core of it? Yeah. Combining information from all parts is again beneficial on the long run*,* I think. [Participant 18]*

### Helping researchers and students

Participants also wanted to share their health data to help researchers and young scientists, as well as training of medical students and other health care professionals in New Zealand.

*That would be fine [sharing health data] as there would be a general benefit for the researchers. [Participant 8]*

*Well*,* I have a daughter who works in the pharmaceutical industry […] providing work for New Zealand trained young scientists too. [Participant 6]*

*I think it comes down to training doctors in hospitals. […] And that*,* in the long run*,* can cut down waiting times and visits to the hospital. [Participant 18]*

*I mean*,* it’s a learning facility and most public hospitals are*,*…you know*,* every little bit helps*,* doesn’t it? [Participant 10]*

### Giving back to the health system

Some participants perceived sharing data as a way to “give back” for health services that they received throughout their lives.

*For me doing this is a way of maybe, in some way, paying back for all the help and all the consideration I’ve had, especially from people and medics at (the) hospital. [Participant 5]*

### Leaving a legacy

Participants perceived their health data as a reflection of their life experiences and personal history, and wanted the collection of these experiences (data) to help others and “do good”, and leave a legacy, even after they died.

*I’m very happy for whatever life experience that I’ve had*,* a bit of negative or positive*,* to be used. […] you know*,* that was painful at the time*,* but it could be used to help somebody - hallelujah*,* you know*,* that’s fantastic! [Participant 23]*

*Well*,* I’m 88*,* and so I’ve not got many years left in my life*,* but […] you’ve got to store this information. It’s not dead. The patient’s not dead. It’s a person. [Participant 22]*

### Good character

The character trait of being caring and helpful was the reason given for why participants were willing to share their own health data. This was also the reason they would wish their data to be used after they were no longer able to consent (either due to dementia or death) so that they might continue to benefit others. Participants also felt that the health information of a family member who had developed dementia should be used if that family member had a caring personality and previously wished to help others.

*My mother developed it (dementia). And she always*,* always said*,* “I’m going to donate my brain to science. If someone can learn from it*,* that’s great’” [Participant 10]*.

*I personally can’t [see a problem with sharing my data] because, as you can see, I’m very open. Yeah, and I’m a health professional myself […] For me the thought that it may help someone is fantastic. [Participant 23]*

### Improve health services

Participants wanted to make systems better and be part of the advancement of systems by sharing their health data. Such systems included the health system, the body of scientific knowledge and the development of new medical treatments. Participants felt that data-driven health services research could cut down hospital visits, improve the way health professionals diagnose illness and lead to more efficiency in the health system.

*Any way that can be helpful for health services I think is great*. *[Participant 16]*

*It [sharing confidential health data] could make the link*,* for better medicine or better care*,* or actions to improve the patient. [Participant 19]*

*It [linking de-identified data with future events] speaks for and promotes efficiency. Yeah. And like a streamlined sort of healthcare system really. [Participant 26]*

### Advance scientific knowledge

Participants wanted their health data to contribute to research and lead to the improvement and advancement of scientific knowledge.

*Without research we don’t go forward. [Participant 18]*

*Any research is good research. I think provided it’s the appropriate medical research as I’d be comfortable with that [sharing health data] [Participant 25]*.

*I think that would be very helpful [linking de-identified information with future events]. Everything that’s gone before ends up with what you’ve got now. [Participant 23]*

### Improving treatments

Participants felt that sharing their health data for research could lead to improvement in treatments and specifically the development of medicines.

*Again*,* anything that can improve medicine in any regard is a good idea. That’s*,* you know*,* how do medicines probably develop over the centuries as it goes? [Participant 19]*

*If it is for the purpose of finding out information that will benefit somebody or help to make a new drug or whatever*,* then I don’t have a problem with that […] I’m happy with that as long as it is to do with improving one’s quality of life*,* or medication. [Participant 3]*

## Concerns

Despite the perceived benefits, participants also had strong concerns about the sharing of their health data. They were apprehensive about sharing data with private companies, sharing inaccurate information, and the personal consequences as well as potential societal consequences.

### Sharing data with private companies: private companies are not trustworthy as custodians of health data

Participants were worried that monetary profit and societal benefit may be mutually exclusive, and therefore did not trust private companies to manage health data. A pervasive theme was the skepticism of how private companies would use health data. Participants were concerned about the intentions of private companies and were worried that their data would be used for the “wrong intentions”: monetary profit and not the benefit of society.

*I don’t trust private companies. I think they’d be using it for profit… Even the big companies make me a little weary. It’s more ethical. You get information on my health*,* it doesn’t really matter*,* but it’s an ethical thing. So think about it: they’re using this to make money. [Participant 7].*

*I’m just thinking*,* well*,* if they’re trying to get profits*,* what are they doing with the profits… bump their money up? [Participant 9]*

*I wouldn’t be so happy about that [sharing data with private companies]. The first thing that runs from my mind is*,* when it’s for profit*,* sometimes money can take away from the whole idea of giving a true perspective. It’s just that if it’s money*,* to do with money*,* maybe it’s the intentions. It’s only about the intentions about if they’re doing it for money. [Participant 3]*

*It’s basically the thing of it being used to make a company money rather than being used to help other people. [Participant 5]*

Even though some participants were strongly opposed to their health data being used for profit, others were strongly supportive of the idea. These participants felt that private companies could use their data for the benefit of society, and specifically to develop new drugs.

*That’s just the world*,* isn’t it? If there’s a company out there*,* they want to make a profit. And if they are making a profit*,* it makes their product better*,* I would say. That’s just how industry works. [Participant 23]*

*It comes down to efforts to help the patients in the future. And companies are there always there to make profits. The most profitable companies in the world are pharmaceutical companies. [Participant 19]*

*No*,* that’s fine because if they’re using it to make a good drug or something then*,* and it’s like make a profit out of it*,* that’s normal society. [Participant 8]*

*A private company has got more time and resources to put into studies*,* I suppose. [Participant 10]*

### Inaccurate information: if health data is inaccurate, it will lead to wrong conclusions

Participants were concerned that the data may be incorrect, and that if such data is shared for research purposes, researchers will come to invalid conclusions.

*Hospitals don’t tend to get the information correct. […] It’s like when I was in (the) hospital*,* I was taken on by ambulance. And they said*,* ’are you a smoker?’ I said no. And then I read in the discharge reports that I was a smoker. […] When people are gathering information like that*,* they see a report that says she was a smoker. […] So the information has to be correct. Because it goes to people like you. And they’re going to say*,* “oh well*,* she did smoke”. And that’s not an accurate result to the research you’re doing. [Participant 18]*

### Consequences: sharing health data may have harmful consequences for individuals and society

Participants were worried that sharing health data may have harmful consequences for the individual whose data is being used, and for society as a whole.

#### Personal consequences

Some were worried about personal implications of sharing their data, such as insurance companies getting hold of the data, or losing their driver’s licence.

*It actually happened to me that my driving license was taken off me […] That’s just an example. If insurance companies go to that*,* they might say “you’re not getting insurance […]*,* you’ve been classified as a drunk driver”. [Participant 4]*

*I worry about the ethics of how will it be used in the future and how will that information*,* yeah*,* affect insurance and all those other things quite ethically…then the people should know about it more. And then it will be a problem. [Participant 11]*

Participants were also concerned about being financially exploited if their private information was shared with private companies.

*I wouldn’t know if I’d be very keen on that… yeah*,* now when they sell your*,* like say your telephone number*,* to these scam people*,* that’s the biggest problem. [Participant 2]*

#### Consequences for society

Some were concerned about the impacts of data sharing on society, such as data being used by governments to control people or even to support warfare.

*Research is often used for warfare and controlling people. And I wouldn’t want that*,* but then how can you stop it? That’s not a reason not to advance scientifically. [Participant 14]*

## Expectations

Participants had strong expectations on how their health data should be used for research, including collaboration to advance scientific knowledge, and data being used in a safe way.

### Collaboration: collaboration between institutions could solve problems in society

Participants were of the opinion that research collaboration between institutions should be strongly encouraged, as it could have many potential benefits and lead to solutions for society as a whole.

*When people work together*,* I think it would be easier for people to find possible questions. Because you’ve got more than one set of advice*,* and everybody’s sharing ideas and having input into it. In that case*,* someone could come up with a solution rather than being in a small pool of people. [Participant 18]*

*The bigger the group*,* the more accurate the result will be. [Participant 19]*

Some participants used the response to the COVID-19 pandemic as an example.

*This is what happened when we had COVID*,* that there was a collaborative work together. Look*,* look*,* we’ve got fantastic vaccines. [Participant 23]*

### Research conduct: research conduct should adhere to ethical principles

Participants had expectations regarding how research should be conducted when using health data. They expected research to be conducted ethically, by adhering to principles such as privacy, transparency and informed consent.

#### Privacy

The main expectation was privacy, and that patients should not be identified at any point.

*As long as they don’t know who I personally am or where I live*,* that’s fine. [Participant 2]*

*I wouldn’t have a problem with it as long as there were no details of myself involved at all. [Participant 18]*

*Well*,* my information is now completely unknown to anybody who’s using it as to who I am*,* so that’s fine. [Participant 8]*

*As long as my privacy is not in danger. I think you said earlier that it will not be known to other people from where this information has been derived. [Participant 1]*

#### Consent

A few participants wanted their consent to be requested before their routinely collected data were linked with other data for research purposes.

*It would be nice to get the consent from the person because obviously they’re going in with the person’s details. So contact the person and say*,* this is what we’re doing. Do you mind if we have consent to your information? Even though down the track you may be de-identified. But still contact the person and say*,* this is what we’re doing. [Participant 18]*

*I think it comes down to the same thing again. If you’re going to do that*,* just ask for consent*,* just say*,* this is what we’re doing for. How do you feel? Yeah. Again*,* it has a lot of benefits…Yeah. Just a courtesy call to say*,* this is what we’re doing. How do you*,* you know*,* we understand you’ve done research in the past. So how do you feel about*,* you know*,* this is what we’re going to do. How do you feel about it? And would you*,* we have me to give consent first and we can all together? Yeah. Just basically like what you’ve done in this interview [Participant 18]*.

Others felt that individual consent was not required (and would be logistically difficult) but they did want to be informed if their health data were likely to be used for research.

#### Transparency

Transparency on what happened to routinely collected health data that was to be used for health research was important for participants.

*If you feel like there’s a huge transparency around it*,* I think you may get public buy-in. That we’re all working to the same good. I mean*,* that might again*,* as I said*,* might be people who were opt out. I mean*,* that we need to have transparency around that”. [Participant 23]*

### Mutual benefit: research should benefit the patients and the institutions that shared health data

There was an expectation that those who use the health data show respect to patients and the institutions that shared the data with them. There was also an expectation that, if the data were shared with private companies or internationally, that the patient group, the hospital and/or the country (New Zealand) would receive some benefit from the research in return, especially if there was monetary gain.

*As long as New Zealand got some benefit back from the research…You know*,* you don’t want to be doing all the work and then half being hijacked from offshore. I don’t know if that makes sort of sense*,* but yeah*,* I’d expect New Zealand to get benefit out of any research like that…that’s been shared. [Participant 19]*

*There’s a lot of people*,* but if the hospital or the*,* you know*,* could make a deal with them (private companies): if you’ve used the research*,* please give us a discount or something. It would go a long way to… You know*,* if there was something where they could make a discount. So many patients could get the benefit of the research or you know*,* maybe somewhere they could work out. So many patients a year for free or whatever. [Participant 4]*

### Cultural respect: health data should be used in a way that respects cultural values

Participants felt that data should be used in a culturally respectful way. Many participants wanted research to be done in a way that was respectful to Indigenous culture in New Zealand. Even though only one of the participants was Māori, there was strong support for Tikanga/ doing research in a way that is appropriate to Māori cultural practices.

*I think actually*,* especially for our tangata whenua*^*1*^, *they might have a problem with that (using data after someone’s death). Because their remains are tapu*^*2*^. *So maybe that is something that should be considered […]. You see*,* I’m okay with that. But other cultures may not be okay with that. [Participant 23]*

## Discussion

Older patients based in the Counties Manukau district of New Zealand were mostly comfortable with their routinely collected health data being used for health research that would benefit their communities, now and in future. Our study demonstrated that older people were strongly supportive of their health data being continued to be used for research if they developed dementia (and were no longer able to consent) or after they had died. Participants felt that people’s previous wishes should be taken into account when deciding whether to include them in research. They said that if people had been willing to help people throughout their lives, it should be assumed that they were also willing to help people even if they had developed dementia (unless they expressly disagreed) and this includes taking part in research. There was a common theme of wanting to give back to the health system for the care they received. The main reasons given were to be able to continue to contribute towards their communities, perhaps reflecting their life stage and experience with the health system.

Participants had strong expectations around the sharing of health data such as deidentification, transparency of processes, privacy and using data in an ethically responsible way. Most strongly supported sharing of deidentified data and collaborative research between health services and academic institutions, however many had a sense of distrust around sharing health data with private companies. A specific consequence that was feared by older adults in our study, was that sharing health data could lead to their driver’s license being taken away, perhaps not surprising in this age group and in participants who are knowledgeable about dementia.

To our knowledge this is the first study to use qualitative research methods to explore older people’s perceptions about sharing of health data. We asked specific questions that prompted people to think about the topics relating to dementia and use of data after death which revealed strong opinions about respecting people’s prior wishes enabling them to continue to contribute to their communities. The responses are rooted in local cultural expectations and are thus an important resource for New Zealanders who may prefer that research is carried out in their own communities rather than depending on the results of research carried out in other populations. The responses reflect those of New Zealanders of mostly European ancestry, so cannot necessarily be generalised to other ethnicities within New Zealand, nor to people living elsewhere. However the attitudes of older patients in New Zealand around sharing routinely collected health data were similar to attitudes expressed by people living in other countries [11, 14, 16], as well as younger New Zealanders, in that participants were mostly positive about the use of their data for research, but opposed the idea of their data being shared with private companies, and feared potential discrimination on the basis of their health data [2, 15, 16].

In our study there was strong support for multi-institution sharing of data to facilitate new discoveries. This finding may have been influenced by the New Zealand experience of the COVID-19 pandemic, where people were very aware of how collaboration led to the discovery of vaccines as well as new variants of the virus. However, the COVID-19 pandemic also made people more aware of how their health data were used by governments to help control disease [2, 13] by controlling the behaviour of the population. Attitudes have changed towards this aspect of disease control and may therefore be the reason for some participants’ concerns about the potential impact on society. Participants in our study were also strongly supportive of their data being used for educational purposes and for young researchers to thrive, however some previous qualitative studies have reported participants being opposed to sharing their health data merely to benefit individual researchers or help advance their careers [15]. This finding may be reflective of the life stage of our participants especially as some mentioned that their children or grandchildren were researchers or students.

Some participants in our study had the expectation that consent should be asked before using routinely collected data for research purposes, although mostly as a “courtesy” rather than an absolute prerequisite. Very few, if any, participants were aware that, alongside other consent procedures on admission to hospital, they also give consent for their data to be used in research and quality improvement projects to improve the health of the local population and local health services. Participants in other studies [15] recognized that the requirement of consent may lead to selection bias and low participation rates. The participants in our study may therefore simply need more information about the consent and research process. We have also identified a need to provide more information to patients about how their data might contribute to improvement of health services and population health outcomes, and to feedback to communities how their data has helped others.

Despite the fact that the majority of our participants were of New Zealand European ethnicity, one of the prominent themes in our study was the consideration for other cultures, specifically Māori culture, and awareness of using data in a culturally respectful way. There was a comparable finding in a study of younger New Zealanders [2] where participants were concerned that if data were shared with international institutions, the overseas researchers may not have a sufficient understanding of Māori culture.

Our study was limited by the high possibility of selection bias. Participants were recruited by asking people who took part in an online survey about whether they would be willing to take part in a one-to-one online interview conducted in English. Recruiting therefore happened from a cohort of people who can be expected to have a relatively high digital literacy. Moreover, interviews were conducted over Zoom, which excluded participants who did not have the hardware, software, or internet connection to participate in such a study. Participants may have found the presented case scenario and accompanying questions challenging to understand, despite the interviewer encouraging opportunities for clarification and discussion. The fact that the majority understood the scenario may corroborate the suggestion that this is a high health literacy sample. The interviews were conducted in English which may have excluded many older non-Europeans who were not comfortable being interviewed in English. In other qualitative research conducted in the same geographic area we found that 80% of Pacific and Asian participants preferred to be interviewed in their native language [20]. Most Māori participants expressed a preference for face-to-face interviews (which are being arranged as part of a future project). This demonstrates the need for more culturally responsive research into the views of other cultures on the sharing of health data.

Another limitation was the case scenario itself. The case scenario was chosen to prompt older participants to discuss their thoughts on sharing routinely collected data. However, the case scenario may have overstated the potential benefits and underestimated the risks associated with data sharing; for example, the risk of re-identification was not specifically mentioned. The questions also did not differentiate between different types of data that participants may be willing to share (for example cognitive versus physical data) and did not capture the complexity of the debate surrounding the early detection and disclosure of dementia risk. These are all important issues as it is likely that, with over 50% of dementia currently unidentified [21], there is increasing enthusiasm to use routinely collected health data to “case-find” dementia. The ethical implications of using health data for dementia case-finding must be addressed [22]. The psychological harm of detecting dementia in a person who did not ask for the assessment might outweigh the benefit of identifying previously undiagnosed dementia [23]. People may not wish to know if they are at risk of a currently untreatable disease, but others may feel that earlier diagnosis gives a person time to plan for the future. These issues are complex, but the use of artificial intelligence with health data is accelerating fast, so it is important that patients and the public are involved in these discussions.

Our participants reinforced the need for governance around data sharing. When routinely collected health data are shared with private companies, this should be done in an ethically responsible way. This becomes pertinent, both now and in future, with the development of Artificial Intelligence (AI). AI could have many potential benefits, especially for the elderly who could benefit from improvements in healthcare. However, it should be developed for the welfare of society, and ethical principles such as transparency, beneficence and privacy should therefore be embedded in its development [8, 24]. This highlights the urgent need for consensus guidelines and legal frameworks to protect health data but also enable its use in a way that can eventually benefit the health system, patients, and society in general.

## Conclusion

The use of older people’s routinely collected health data for health research may lead to potential benefits for this age group. Older people in our study were supportive of sharing their health data for research purposes provided that the research would benefit others, now and in the future. They expressed a desire to be included and to continue contributing to society as they get older and also after they have died. However, they expressed concerns about the consequences of sharing data, and they had strong expectations on how the data should be used in an ethical and responsible manner. The research highlights the obligation of researchers to handle data in a culturally respectful way and to use it to benefit communities while avoiding potential harms.

## Electronic supplementary material

Below is the link to the electronic supplementary material.

Additional_file_1: Title of data: Individual Interview Scenario. Description of data: Topic guide for the semi-structured interview.

## Data Availability

The datasets generated and/or analysed during the current study are not publicly available due high risk of participants identification but are available from the corresponding author on reasonable request.
