# Supplementary material for: Contribution and legacy: a qualitative study of older people’s attitudes about sharing their routinely collected health data for research purposes in New Zealand
Source: BMC Med Ethics. 2025 May 30;26:70. doi: 10.1186/s12910-025-01212-6 (PMC12124040; doi:10.1186/s12910-025-01212-6)
Supplement: Supplementary file 1 — Additional_file_1: Title of data: Individual Interview Scenario. Description of data: Topic guide for the semi-structured interview. [file 12910_2025_1212_MOESM1_ESM.docx]

**INDIVIDUAL INTERVIEW SCENARIO**

**Information for the interviewer:** The scenario is based on creating a clinical risk tool and covers different areas of concern e.g., de-identified health information, data linkage, disease registries, using health information from the deceased, working with academics and/or other institutions inside NZ and outside NZ. Participants will be presented with the different stages of the scenario and prompted to discuss their thoughts on each stage including thoughts about the use of their de-identified health information in the way described in the stage and any concerns or parts they may be uncomfortable with. We start with a review of how health information is used, then go onto the scenario that takes the use of de-identified health information into the realm of research and/or commercialisation.

**CONSENT PROCESS (either written or recorded verbal)**

**INTERVIEW:**

**I am going to give you some information about how Middlemore Hospital might currently use your health information, and then discuss ways in which they might use your health information for research. If at any time you do not understand what has been said, please tell me and I will explain.**

**Are you happy for us to begin?**

**First of all, I am going to tell you how your personal health information is currently used:**

Clinicians use a person's individual health information to guide their current treatment and care. For example, they might look at a person’s blood tests and CT scans to help decide about a diagnosis and whether someone needs further care and support. They share the information with other people who look after that person’s healthcare such as their GP or clinicians at another hospital.

When we analyse health information from a larger group of people (for example all people using older adult services at Middlemore Hospital) we can create statistics which are used to ensure that the hospital services are running smoothly (for example, waiting times). Health information used in this way is “de-identified”. De-identified means that any identifying information such as your name, address, date of birth, NHI number is removed so that just the health information is left. No-one can tell which health information belongs to which individual after de-identification.

**In this interview we are interested in how de-identified health information can be used in other ways, e.g., for research.**

**I am going to give you some more information and then ask you for your opinion about it. If at any time you do not understand what has been said, please tell me and I will explain.**

Decline in brain health is partly due to the same risk factors that affect the body, e.g., diabetes, high blood pressure, being overweight, high cholesterol. A group of clinicians and researchers want to develop a tool that helps to identify people at higher risk of decline in brain health. The benefit of this is that clinicians can intervene early to reduce the chances of decline in brain health and/or dementia.

To do this they first need to create a register of a large group (over 2000) of patients aged 55 and over who have attended an outpatient clinic, some will have memory problems (poor brain health) and some will not. The register will hold some of their health information for example their previous and current diseases, blood tests, CT scan reports, and cognitive tests if they have been done.

The health information in the register is then “de-identified”. De-identified means that any identifying information such as your name, address, date of birth, NHI number is removed so that just the health information is left. No-one can tell which health information belongs to which individual after de-identification.

The de-identified health information from the whole group can be analysed to find out what past health information might predict people who are at higher risk of decline in brain health. This would provide better information to us about how people can protect their brain health. The analysis of de-identified health information would therefore be for the benefit of others rather than the individual whose health information has been used.

**QUESTIONS:**

1. Imagine that your health information has been selected to be de-identified and analysed as part of the large group of 2000 people described above. No-one can tell which information belongs to you after de-identification.

How do you feel about your de-identified health information being used in this way?

1. The use of de-identified health information is currently used for service improvement and research. The de-identified health information is sometimes also shared with researchers (eg from the University of Auckland) who can do more complex analysis to answer more complex questions.

How do you feel about sharing your de-identified health information with researchers or clinicians from partner institutions in NZ (e.g., University of Auckland) to collaborate on the research or do the analysis?

1. Sometimes the findings are more accurate if there is a larger sample. So, researchers share their de-identified health information with other universities who have similar de-identified health information and are doing similar research to get a bigger sample and more precise results.
2. How do you feel about sharing your de-identified health information with other universities in NZ?
3. Follow-up question: How about universities outside NZ (e.g., Australia) and therefore a non-New Zealander interpreting the health information?
4. Follow-up question: What about universities in the UK or USA?
5. Sometimes researchers will link the health information to see if different factors are related, for example an individual’s diabetes blood tests and their CT brain scan. The health information is still de-identified so that no-one can tell which information belongs to which individual.

How do you feel about your de-identified health information being linked to find out if different factors are related?

1. Sometimes researchers will link the health information with future events such as outpatient visits and inpatient stays, moving into a care home. The health information is still de-identified so that no-one can tell which information belongs to which individual.

How do you feel about your de-identified health information being linked with future events such as outpatient visits and inpatient stays, or if you had moved into a care home.

1. If lots of people’s de-identified health information (for example brain scans) are analysed together it can be used to develop computer programmes that can read the scans of people who have the same brain scans in the future, making diagnosis of brain diseases more accurate, quicker and cheaper than traditional methods.
2. How do you feel about researchers using your de-identified health information to create a computer programme only for use in the local hospital?
3. What about if it was used in other NZ hospitals?
4. How do you feel about companies using your de-identified health information to create computer programmes for other health providers (e.g., another hospital in another country)?
5. How do you feel about companies using your de-identified health information to create and sell a computer programme to other health providers to make a profit?
6. It is important to be able to use de-identified health information from all patients including those who have died, otherwise the health information is only from the most healthy people and will produce inaccurate results.

How do you feel about sharing the use of de-identified health information from all relevant patients (including deceased)?

1. We have talked about researchers using de-identified health information from a large group of patients to look at relationships between different factors e.g., diabetes and brain scans; about relationships with future events e.g., moving into a care home; and using health information to create computer programmes that help with diagnosis. We have talked about the use of such information by local clinicians and researchers, by other health researchers inside and outside NZ and by commercial companies.

You have answered questions about your own health information being used in this way. How would you feel (would you feel different) if your whānau/family members’ de-identified health information was used in the way same way as described above? (Interviewer might need to prompt reminders)

1. This is a summary of your answers to your questions (summarise their answers for questions 1-8).

Please let me know if there is anything else that you want to tell me or you think that I might have missed. Have any of your answers changed while we have been talking?

**Thank you for your time today – your contribution is much appreciated.**
